# Hypoxia-Induced miR-675-5p Supports β-Catenin Nuclear Localization by Regulating GSK3-β  Activity in Colorectal Cancer Cell Lines

**DOI:** 10.3390/ijms21113832

**Published:** 2020-05-28

**Authors:** Laura Saieva, Maria Magdalena Barreca, Chiara Zichittella, Maria Giulia Prado, Marco Tripodi, Riccardo Alessandro, Alice Conigliaro

**Affiliations:** 1 Department of Biomedicine, Neuroscience and Advanced Diagnostics (Bi.N.D.), Section of Biology and Genetics, University of Palermo, 90133 Palermo, Italy; laura.saieva@unipa.it (L.S.); mariamagdalena.barreca@unipa.it (M.M.B.); chiara.zichittella@community.unipa.it (C.Z.); riccardo.alessandro@unipa.it (R.A.); 2 Istituto Pasteur-Fondazione Cenci Bolognetti, Department of Molecular Medicine, Sapienza University of Rome, 00185 Rome Italy; mariagiulia.prado@uniroma1.it (M.G.P.); marco.tripodi@uniroma1.it (M.T.); 3 National Institute for Infectious Diseases L. Spallanzani, IRCCS, 00161 Rome, Italy

**Keywords:** hypoxia, miR-675, long non-coding H19, colorectal cancer, β-catenin

## Abstract

The reduction of oxygen partial pressure in growing tumors triggers numerous survival strategies driven by the transcription factor complex HIF1 (Hypoxia Inducible Factor-1). Recent evidence revealed that HIF1 promotes rapid and effective phenotypic changes through the induction of non-coding RNAs, whose contribution has not yet been fully described. Here we investigated the role of the hypoxia-induced, long non-coding RNA H19 (lncH19) and its intragenic miRNA (miR-675-5p) into HIF1-Wnt crosstalk. During hypoxic stimulation, colorectal cancer cell lines up-regulated the levels of both the lncH19 and its intragenic miR-675-5p. Loss of expression experiments revealed that miR-675-5p inhibition, in hypoxic cells, hampered β-catenin nuclear localization and its transcriptional activity, while lncH19 silencing did not induce the same effects. Interestingly, our data revealed that miRNA inhibition in hypoxic cells restored the activity of Glycogen Synthase Kinase 3β (GSK-3β) reducing the amount of P-Ser9 kinase, thus unveiling a role of the miR-675-5p in controlling GSK-3β activity. Bioinformatics analyses highlighted the serine/threonine-protein phosphatases PPP2CA, responsible for GSK-3β activation, among the miR-675-5p targets, thus indicating the molecular mediator through which miR-675-5p may control β-catenin nuclear localization. In conclusion, here we demonstrated that the inhibition of the hypoxia-induced non-coding RNA miR-675-5p hampered the nuclear localization of β-catenin by regulating GSK-3β activity, thus proposing the miR-675-5p as a new therapeutic target for the treatment of colorectal cancer.

## 1. Introduction

Colorectal cancer (CRC) is a heterogeneous disease with an environmental, genetic and biochemical background. It is the third most commonly diagnosed cancer and the fourth in terms of cancer-related deaths, and it is expected to increase worldwide by 60% with more than 2.2 million new cases and 1.1 million cancer deaths by 2030 [1]. Mutations in Adenomatous Polyposis Coli (APC) gene and/or its hyper-methylation are events common to ~80% of sporadic colorectal tumors and often represent the driver event for further occurrence of mutations. APC constitutes, together with Axin, Glycogen synthase kinase 3β (GSK-3β), and Casein kinase 1 (CK1), the “degradation complex”, a multiprotein complex responsible for β-catenin phosphorylation, poly-ubiquitination and subsequent degradation through proteasome [2]. The binding of canonical Wnt ligand to its receptors inactivates the degradation complex, allowing for β-catenin nuclear translocation and the induction of its targets in cooperation with the TCF/LEF transcription factors. In CRC, Wnt signaling dysregulation is associated with proliferation, invasion, differentiation, and cell resistance both in early stages as well as during cancer progression [3]. 

Even if Wnt//β-catenin pathway dysregulation is most often due to APC mutations, it is also reported that approximately 1% of CRC patients with wild-type APC have activating mutations in the β-catenin protein [4]. Interestingly, APC and β-catenin mutations are mutually exclusive events and associate with different types of colorectal tumors [5]. 

The accumulation of mutations in one or more genes promotes the onset and progression of cancer; meanwhile, the molecular alterations in the tumor microenvironment endorse its aggressive phenotype. Within a growing neoplastic mass, the reduction of oxygen partial pressure and the activation of hypoxic responses are among the drivers for the CRC metastasizing process [6]. The rapid growth of the primary tumor is not sufficiently supported by a concomitant development of the vascular system; this determines a reduction of oxygen partial pressure in the tumor microenvironment, and the stabilization of the transcription factor HIF-1α (Hypoxia Inducible Factor-1α), that, accumulated in the nucleus, will form a stable complex with the HIF-1β subunit. The binding of HIF1 complex to genes with functional hypoxia response elements (HREs) induces, in hypoxic cells, the activation of numerous survival strategies, i.e., the metabolic reprogramming, the maintenance of stemness, motility, invasion, angiogenesis, immune suppression, resistance to apoptosis and chemoresistance [7]. These phenotypic transformations are ascribable to the large number of HIF1 targets, among which have been counted more than 70 coding-genes, and to which have been recently added numerous non-coding RNAs that support HIF1 activity both at epigenetic and post-transcription level [8,9,10,11]. Functional HRE elements have been found in the promoter region of the long non-coding RNA H19 (lncH19), and oncogenic roles have been attributed to hypoxia-induced lncH19 [12,13]. Moreover, it has been demonstrated that in the hypoxic tumor, together with the increase of lncH19, there is a selective increase of miR-675-5p, one of the two microRNAs (miRNAs) located in the first exon of the long non-coding [14,15,16]. In glioblastoma, miR-675-5p is necessary for maintaining hypoxic responses by controlling the HIF1α mRNA stability [15] and, in CRC, it participates in tumor progression by regulating HIF1α-induced EMT (epithelial-mesenchymal transition) [14]. Recently, growing evidence demonstrated that Wnt and HIF pathways synergize in promoting the tumor growth [17,18,19,20,21,22]. With the aim to identify molecular mechanisms through which the two pathways could reinforce each other, in this study, we investigated the role of hypoxia-induced non-coding RNAs (lncH19 and miR-675-5p) on two different CRC cell lines: the SW620 and the HCT116, that display, respectively, mutation in APC and β-catenin [5,23,24].

## 2. Results

### 2.1. Hypoxia Upregulates the Expression of Both miR-675-5p and lncH19 in CRC Cell Lines with Mutated Wnt Pathway Components

**Figure 1:**
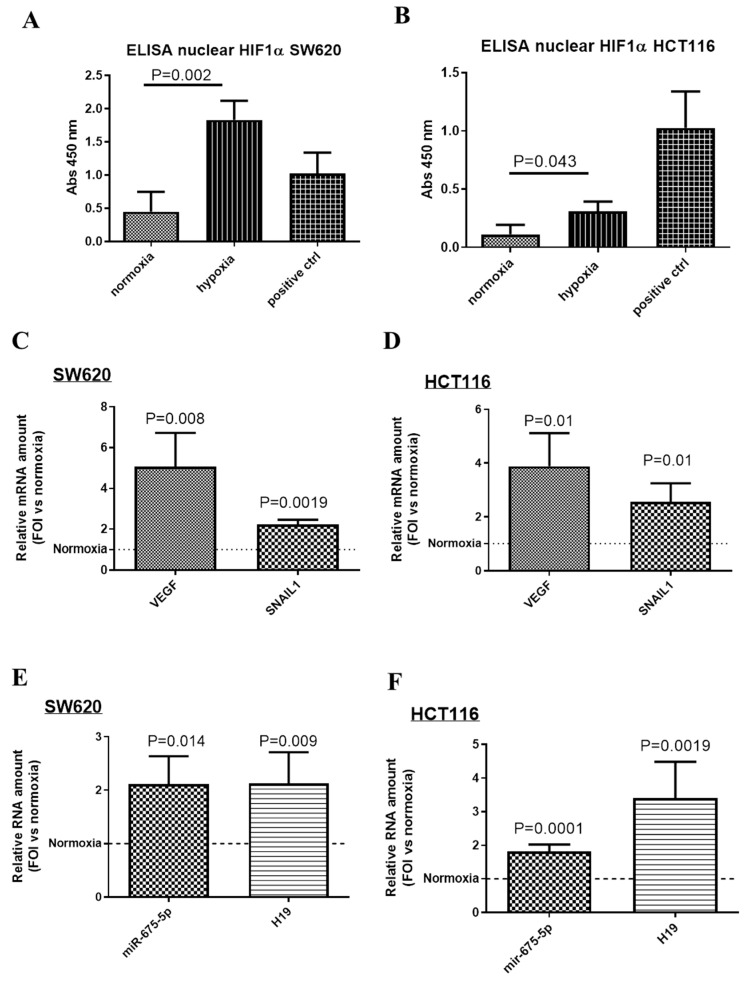
CRC cell lines up-regulated miR-675-5p and lncH19 after 18 h of hypoxic stimulation. ELISA assay for HIF-1α performed with nuclear extracts of SW620 (**A**) and HCT116 (**B**) after 18 h of hypoxic stimulation, positive control was furnished by the kit and it is the nuclear extract of HeLa cells pretreated with LiCl. Data, expressed as absorbance (ABS) values at 450 nm, are the mean ± SD of three independent experiments. Real-time PCR for VEGF, SAIL1, miR-675-5p and lncH19 in SW620 (**C**,**E**) and HCT116 (**D**,**F**) stimulated for 18 h in the hypoxic chamber. MiR-675-5p data were normalized for RNU6 (RNA, U6 Small Nuclear 1) while VEGF, SNAIL1, and lncH19 levels were normalized for β-actin, ΔΔCt is expressed as fold of increase (FOI) with respect to normoxic conditions. Data are expressed as the mean ± SD of three independent experiments and *p*-values are indicated in the graph.

In order to investigate the role of early-induced non-coding RNA during hypoxic stimulation, CRC cell lines SW620 and HCT116 underwent hypoxic chamber stimulation for 18 h. Both cell lines physiologically respond to low O_2_ partial pressure stimulation by increasing the amount of nuclear HIF1α transcription factor as indicated by the ELISA assay in Figure 1A,B. Moreover, HIF1α transcriptional activity was confirmed by the increase in the expression levels of two of its targets, VEGF and SNAIL1 [25,26], in respect to their levels in normoxic conditions (Figure 1C,D).

As previously demonstrated for SW620 cells [14] and here confirmed also for HCT116 cells, hypoxic stimulation induced also an increase in the transcription of two non-coding RNAs: the long non-coding RNA H19 (lncH19) and its intragenic miRNA miR-675-5p (Figure 1E,F). The correlation among lncH19 and CRC has been widely investigated [27,28,29,30] as well as its crosstalk with Wnt pathway [31] while not much is known about the role of hypoxia-induced miR-675-5p in colon cancer. 

**Figure 2:**
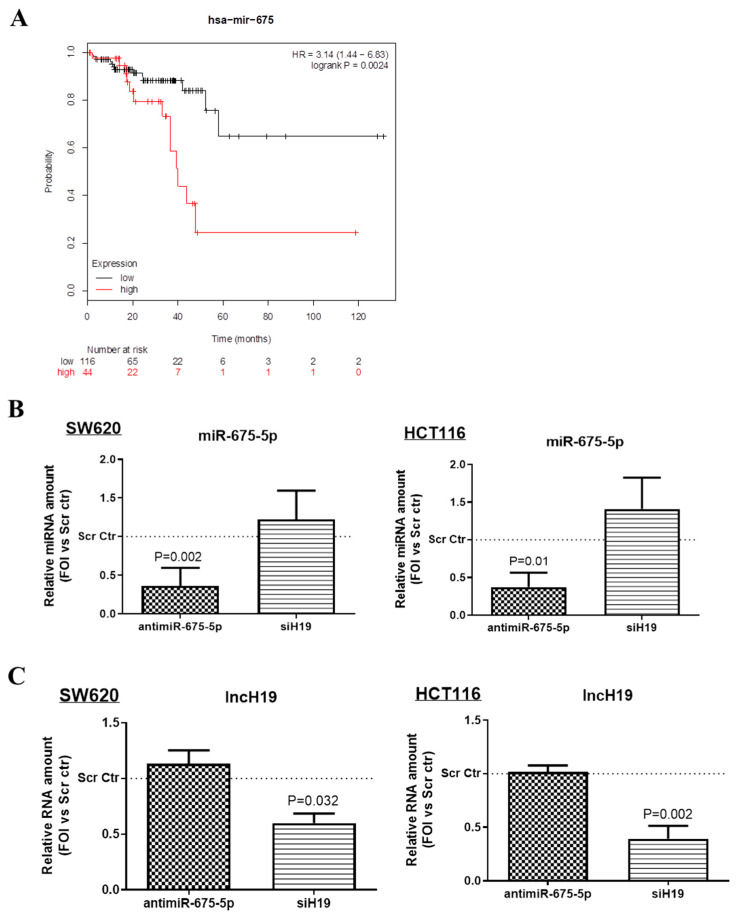
MiR-675-5p regulation (**A**) Kaplan–Meier curve for overall survival in the cohort of 160 rectal cancer patients. The plot was drawn by using the online Kaplan–Meier plotter tool. Patients were divided into low and high expression groups based on an upper-tertile cut off value of 20. (**B**) Real-time PCR for miR-675-5p in SW620 (on the left) and HCT116 (on the right) transfected with AntimiR-675-5p, siH19 or relative scrambled negative control and subjected to 18 h of hypoxic stimulation. (**C**) Real-time PCR for lncH19 in SW620 (on the left) and HCT116 (on the right) transfected with AntimiR-675-5p, siH19 or relative scrambled negative control and subjected to 18 h of hypoxic stimulation. MiR-675-5p data were normalized for RNU6 (RNA, U6 Small Nuclear) while lncH19 levels were normalized for β-actin, ΔΔCt is expressed as fold of increase (FOI) with respect to scrambled negative control. Data are expressed as the mean ± SD of three independent experiments and *p*-values are indicated in the graph.

Bioinformatics analysis performed by the Kaplan-Meier plotter online tool, shown in Figure 2A, suggested that high levels of miR-675 are associated with a poor prognosis in rectal cancer (*n* = 160, *p* < 0.05). Moreover, the analysis of miR-675 targeted pathways, conducted by the use of miRWalk2.0 [32], indicate a significative interaction among miR-675 and both Wnt pathway genes and CRC associated genes (Table S1).

These data prompted us to investigate if the hypoxia-induced non-coding RNAs participate in the dysregulating β-catenin activity.

### 2.2. MiR-675-5p Controls Beta Catenin Nuclear Translocation 

In order to identify the specific role of miR-675-5p and/or lncH19 in hypoxic cells, CRC cell lines were transfected with miR-675-5p inhibitor or silenced for lncH19 expression before undergoing the hypoxic stimulus. The efficiency of the inhibition is shown in Figure 2B,C. Although it is widely assumed that miR-675-5p is obtained by the processing of lncH19, it is to be noted that in hypoxia, the silencing of lncH19 did not affect miR-675-5p expression levels, the same occurred for miR-675-p inhibition against H19 expression levels.

**Figure 3:**
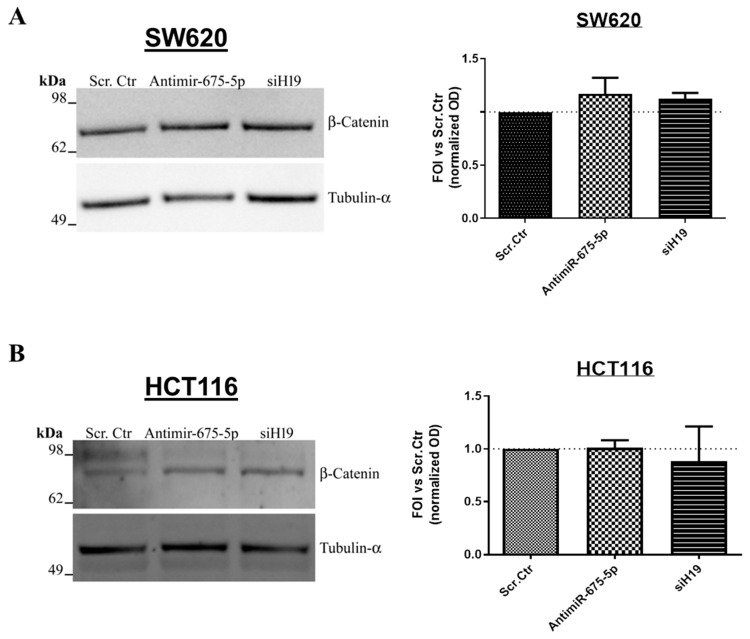

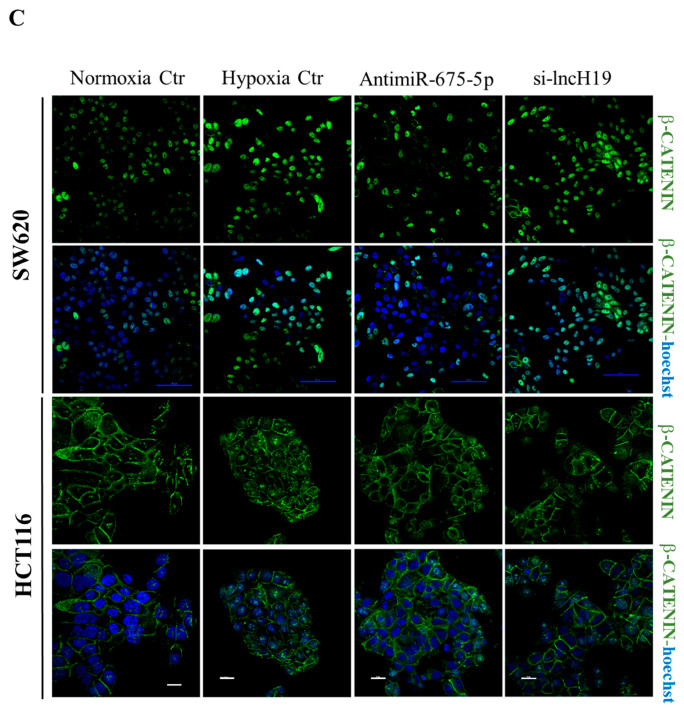
Effects induced on β-catenin by miR-675-5p inhibition or lncH19 silencing. Representative images and densitometric analyses of the Western blots for β-catenin on total extract proteins from SW620 (**A**) and HCT116 (**B**) transfected with AntimiR-675-5p, siH19 or scrambled negative control and subjected to 18 h of hypoxic stimulation. Data are expressed as the mean ± SD of three independent experiments. (**C**) Immunofluorescence for β-catenin on SW620 (upper panels) and HCT116 (lower panels) in the different culture conditions. Normoxic cells transfected with scrambled negative control, hypoxic cells transfected with AntimiR-675-5p, siH19 or relative scrambled negative control. β-catenin in green, Hoechst stained nuclei in blue. The blue scale bar is 50 µm (in SW620) while the white one is 20 µm (in HCT116).

Firstly, we investigated β-catenin protein levels in CRC cell lines inhibited for the expression of miR-675-5p (antimiR-675-5p) or for lncH19 (siH19) and undergone to hypoxic stimulation. As shown by the Western blot in Figure 3A,B the non-coding RNAs’ inhibition did not affect the protein amounts in both cell lines. It has been largely described that hypoxic stimulation promotes β-catenin nuclear localization also in CRC cells which, already in normoxia, showed β-catenin nuclear activity [33,34,35]. Interestingly, the immunofluorescence analyses, in Figure 3C, revealed that miR-675-5p inhibition reduced the hypoxia-induced nuclear accumulation of β-catenin in both cell lines while the lncH19 silencing did not show the same effects. The trend of the nuclear reduction is confirmed by the Western blots in Figure S1 (SF1).

**Figure 4:**
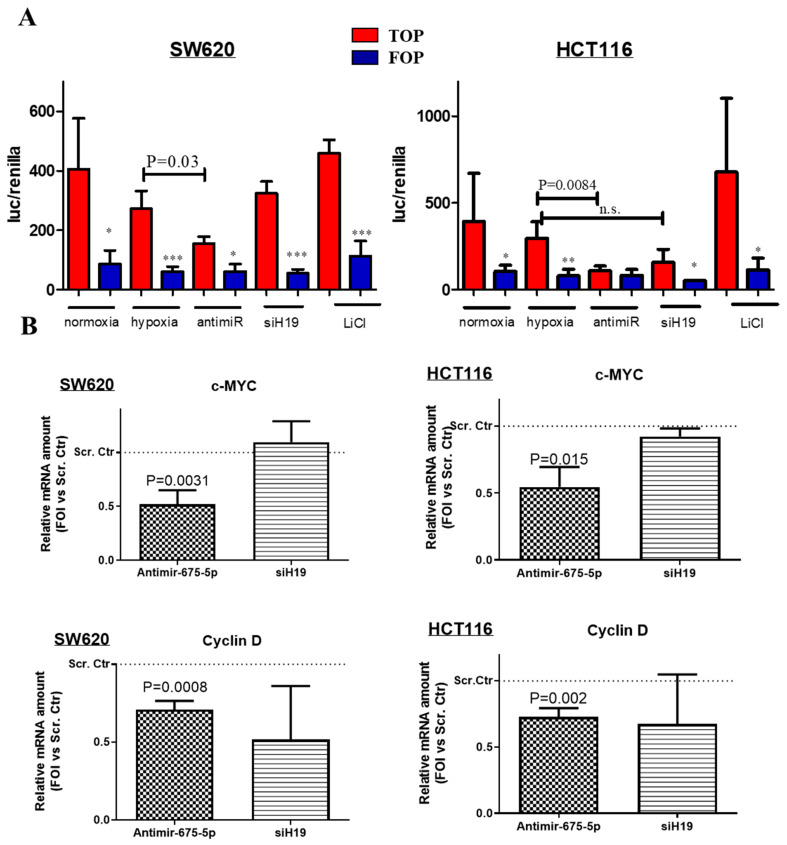
Effects induced on β-catenin activity by miR-675-5p inhibition or lncH19 silencing in hypoxic cells. (**A**) Dual Glo luciferase assay on SW620 (left graph) and HCT116 (right graph) transfected with TOP- Flash or FOP-Flash plasmid in normoxic condition or co-transfected with AntimiR-675-5p, siH19 or scrambled negative control, and subjected to 18 h of hypoxic stimulation. Normoxic cells transfected with TOP-Flash or FOP-Flash plasmid and treated with LiCl were used as positive control of the assay. Data are expressed as the mean ± SD of three independent experiments. The *p*-values of the differences between TOP vectors are indicated in the graph, while the * indicates the *p*-values of the difference between TOP and FOP for each condition. * < 0.05, ** < 0.005, *** < 0.0005. (**B**) Real-time PCR for c-MYC and Cyclin D1 in SW620 (left panels) and HCT116 (right panels) transfected with AntimiR-675-5p, siH19 or relative scrambled negative control and subjected to 18 h of hypoxic stimulation. Gene expression data were normalized for β-actin, ΔΔct is expressed as fold of increase (FOI) with respect to the expression in the scrambled negative control transfected cells. Data are expressed as the mean ± SD of three independent experiments and *p*-values are indicated in the graph.

Then, we investigated the effects on β-catenin transcriptional activity induced by miR-675-5p and/or lncH19 silencing in hypoxic cells. To this aim, we transfected cells with the TOP-Flash FOP-Flash vectors that contain consensus TCF/LEF binding element (respectively, wild type or mutated) upstream of luciferase expression construct. The graphs in Figure 4A demonstrated that the transcriptional activity of β-catenin on TCF/LEF promoter is affected by miR-675-5p inhibition in hypoxic cells. As expected, no variation in the luciferase activity was revealed in response to the different treatments in cells transfected with the mutated promoter (FOP). Further confirmation was obtained by the transcriptional analysis for the β-catenin targets c-MYC and Cyclin D1, involved in regulating tumor growth and cellular proliferation. As shown by the RT-PCR graphs in Figure 4B, miR-675-5p inhibition reduced both c-MYC and Cyclin D1 expression in hypoxic CRC cell lines. While c-Myc is known to be down-regulated also by HIF complex in prolonged hypoxia [36,37], it is noteworthy the inhibitory effect induced by miR-675-5p on Cyclin D1 expression, which we found up-regulated in hypoxic CRC cells as shown in Supplementary Figure S2 (SF2).

### 2.3. MiR-675-5p Inhibition in Hypoxic Cells Promotes GSK3β Activation 

To identify the molecular mediators by which miR-675-5p may control β-catenin nuclear translocation, we focused on the Wnt pathway genes that showed miR-675-5p target sequences on their transcripts, and in particular, on the Ser/Thr kinases GSK3β, the core protein of the “destruction complex” which phosphorylates β-catenin at Ser33 and Ser37 residues [38].

MiRWalk2.0 [32] analysis (Table S2) identified consensus sequences for miR-675-5p on GSK3β, but also in the three serine/threonine-protein phosphatases (PPP2CA, PPP2R1A, PPP2R2B) that form the PP2Ca_R1A_Bd complex. The latter controls GSK3β activation by Ser9 residue de-phosphorylation, as shown in SIGNOR 2.0 database [39], a database of causative relationships between biological entities coming from scientific literature (https://signor.uniroma2.it/relation_result.php?id=SIGNOR-C133&organism=human). 

**Figure 5:**
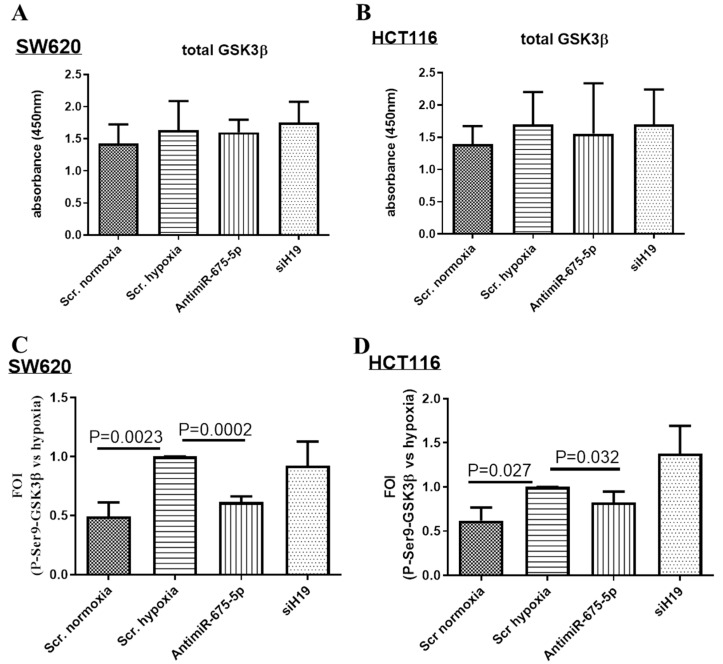
MiR-675-5p inhibition favors GSK3β activation in hypoxic cells. Total GSK3β assay in SW620 (**A**) and HCT116 (**B**) transfected with AntimiR-675-5p, siH19 or relative negative control and subjected to 18 h of hypoxic stimulation, or transfected with the scrambled negative control and maintained in normoxic condition. Data are expressed as absorbance (ABS) values at 450 nm, and are represented as the mean ± SD of three independent experiments. Total/P-Ser9-GSK3β ELISA assay in SW620 (**C**) and HCT116 (**D**) treated as described above. Data of P-Ser9-GSK3β were normalized for total GSK3β and expressed as fold of increase (FOI) with respect to the P-Ser9 GSK3β levels in hypoxic cells transfected with scrambled negative control. Data are expressed as the mean ± SD of three independent experiments and *p*-values indicated in the graph.

First, we evaluated the effects of miR-675-5p inhibition on GSK3β. By the use of ELISA assay, we found that the total amount of GSK3β was not affected by hypoxic stimulation, either by miR-675-5p inhibition or by lncH19 silencing (Figure 5A,B). Intriguingly, evaluating the inactive P-Ser9-GSK3β, we found that the hypoxic stimulus had an inhibitory effect on the GSK3β activity, but this inhibition was lost when the cells were transfected with antimiR-675-5p; these results indicated that miR-675-5p has a role in maintaining GSK3β inactive in hypoxic cells. In Figure 5C,D, the amount of inactive P-Ser9-GSK3β was normalized for the total amount of GSK3β in the different conditions, and reported as fold of induction versus the equivalent in hypoxic controls. 

**Figure 6:**
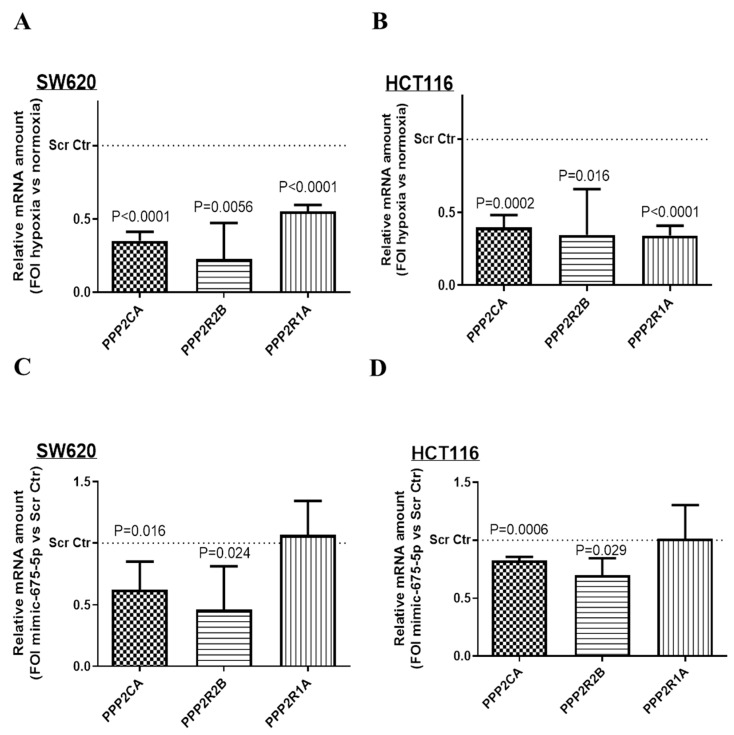
Effect of miR-675-5p on the phosphatases’ mRNA level. Real-time PCR for PP2CA, PP2R2B and PP2R1A in SW620 (**A**) and HCT116 (**B**) under normoxic condition or subjected to 18 h of hypoxic stimulation. Gene expression data were normalized for β-actin, ΔΔct is expressed as fold of increase (FOI) with respect to the expression in normoxic cells. Real-time PCR PP2CA, PP2R2B and PP2R1A in SW620 (**C**) and HCT116 (**D**) transfected with miR-675-5p mimic or scrambled negative control in normoxic conditions and subjected to 18 h of hypoxic stimulation. Gene expression data were normalized for β-actin, ΔΔct is expressed as fold of increase (FOI) with respect to the expression in scrambled negative control transfected cells. Data are expressed as the mean ± SD of three independent experiments and *p*-values are indicated in the graph.

Then, we moved to validate the three serine/threonine-protein phosphatases of the PP2Ca_R1A-Bd as miR-675-5p targets. The transcriptional analysis of the three serine/threonine-protein phosphatases (PPP2CA, PPP2R1A, PPP2R2B) in Figure 6A,B showed that hypoxic stimulation strongly down-regulated the mRNA levels of all of them. Then, to investigate if this inhibitory effect might be due to miR-675-5p hypoxia-induced over-expression, cells were transfected with miR-675-5p mimic or scrambled control under normoxic condition, and phosphatases’ RNA levels were analyzed by real-time PCR. As shown in Figure 6C,D, miR-675-5p mimic, in both cell lines, reduced RNA levels for both PPP2CA and PPP2R2B, respectively, and the catalytic subunit and the B regulatory subunit of the serine/threonine-protein phosphatase 2A, while no effects were had on PPP2R1A mRNA amount; however, we cannot exclude an effect of miR-675-5p on the phosphatases’ translation levels.

## 3. Discussion

In this study, by the use of two CRC cell lines mutated in APC and β-catenin, we demonstrated that the aberrant activation of β-catenin is further supported by hypoxia-induced non-coding RNA activity/modulation. In particular, we found that in hypoxic cells, the induction of miR-675-5p endorsed β-catenin nuclear localization by affecting GSK3β activity, thus identifying in the miR-675-5p, a putative therapeutic target to inhibit aggressive CRC.

In the last few years, the increasing amount of information acquired about the biology of cancer cells and the molecular mechanisms that govern the onset and progression of the tumor has allowed the development of new drugs [40,41]. However, the high mortality rate in subjects with CRC and the increased risk of metastases, including distant bone metastases with subsequent complications such as pain and pathological fractures due to bone destruction [42,43], indicate the need of more effective and targeted therapies, especially for the treatment of metastatic colon cancer. 

In CRC, lncH19 is considered among the oncogenic long non-coding RNAs [29]. Its role in promoting the growth and progression of cancer has been traced back to its ability to control gene expression by interacting with several proteins, including transcription factors, chromatin regulating proteins, and RNA binding proteins. Its pro-oncogenic role has also been attributed to the ability to sponge several microRNAs with a tumor suppressor role for CRC, e.g., let-7 and miR200 family [44,45].

Moreover, the lncH19 is the precursor of miR-675, recently found involved in physiological processes and cancer development, including CRC [14,46,47]. 

Intragenic miRNAs are largely co-transcribed, and consequently co-regulated with their host genes. Furthermore, functional linkages between intragenic miRNAs and their hosts on multiple levels have been identified [48,49,50]. However, not much is known about the regulation of miR-675; this is generally associated with lncH19 expression and often considered, by the literature, to be among the mechanisms of action of the lncH19 itself. Such considerations could lead to believe lncH19 silencing as an effective strategy to stop cancer progression; this in order to inhibit both the long non-coding and the miR-675. Our data demonstrated that this strategy would not be successful, at least in the treatment of CRC.

Notably, we found that the hypoxia-induced miR-675-5p expression was not abolished by lncH19 silencing, thus releasing, at least in part, the synthesis of the miR-675-5p from the long non-coding that cages it. Moreover, in our system, miR-675-5p inhibition in hypoxic cells is most effective than lncH19 silencing to inhibit β-catenin nuclear activity; the latter characteristic common to 80% of CRCs [51]. 

Here, we have highlighted a new role for hypoxia-induced miR-675-5p in controlling the β-catenin nuclear localization in hypoxic cells, with effects that could go well beyond the investigation carried out so far. In this study, in fact, we focused our interest on the β-catenin targets identified by the consensus TCF/LEF binding elements, which are expressed also in normoxic condition, due to the common Wnt pathway mutations in CRCs. 

However, it is known that during hypoxia, nuclear β-catenin is recruited also on HREs to cooperate physically with HIF1 in activating some of its targets, promoting cell survival and adaptation to hypoxia [52,53]. Therefore, further studies may reveal that the failure to recruit β-catenin could broadly affect the hypoxic response, confirming the key role of miR-675 in supporting hypoxic CRC as already demonstrated for glioblastoma [15]. 

In addition, our data revealed that the miR-675-5p acts on the nuclear localization of the β-catenin without affecting the total amount of the protein. Interestingly, in colon cancer, a role of β-catenin at the cytoplasmic level has emerged where, interacting with other RNA binding proteins, it participates in the stabilization of several mRNA [54]. Furthermore, it was demonstrated that the cytoplasmic accumulation of β-catenin, in response to hypoxia, activates a post-transcriptional de-differentiation and survival program in breast cancer cells [55]. Further efforts should be directed to identifying all the β-catenin cytoplasmic targets to evaluate whether they are affected in response to hypoxia or to miR-675-5p inhibition. 

The use of miR-675-5p as a therapeutic target for the inhibition of β-catenin nuclear localization would have an effect in CRC cells where the APC-mediated GSK3-β-dependent regulation of β-catenin is functional. Here, it is to note that even if APC mutation was found in 80%–90% of both inherited and sporadic colorectal cancers, as reviewed by [56], APC-mutated CRCs usually do not carry homozygous null mutations but keep at least one allele encoding a truncated APC protein that retains a residual ability to target β-catenin for degradation. This makes the WNT pathway inhibition a viable strategy for the treatment of the majority of CRCs. We cannot exclude that miR-675-5p inhibition may also have a role in normoxic cells but further studies are required to investigate this point since normoxic cells present a cellular and molecular scenario completely different from that one in hypoxia [57,58,59].

Furthermore, our data demonstrated that miR-675-5p affects GSK3β activity in hypoxic cells, and although the study focused on β-catenin, it opens the door to additional investigation considering that GSK3β has regulatory roles in several cellular processes, being the “molecular hub” that connects pathways responsible for tumor invasion and chemoresistance [60]. Finally, as a putative mechanism of action for the miR-675-5p, we identified the inhibition of PP2CA, the catalytic subunit of the PP2A phosphates [61]. This is considered to be a principal guardian against tumorigenic transformation [61,62] and, interestingly, the evidence from literature indicated that PP2A inhibition is a common event in several cancers including CRC [63,64]. 

## 4. Materials and Methods

### 4.1. Cell Culture

SW620 and HCT116 cells obtained from DBA were cultured respectively in RPMI (Euroclone, UK) and in McCoy’s 5A medium (Life Technologies; Carlsbad, CA, USA), supplemented with 10% fetal bovine serum, 1% penicillin/streptomycin (50 IU/mL) and 2mM glutamine (Life Technologies). Cells were maintained in a humidified 5% of CO_2_ atmosphere at 37  °C. For all the experiments, cells were used at an early passage. For hypoxia experiments, both cell lines were seeded at 50.000 cells/cm^2^ and were incubated in a hypoxia chamber containing 1% O_2_ gas mixture for 18 h. 

### 4.2. Transfections

Cells were transfected by using Lipofectamine 3000 reagent (cat. L3000-015, Life Technologies) following manufacturer’s standard protocol. SW620 and HCT116 cells were seeded at 50.000 cells/cm^2^ in a 12-well plate, and transfected with 30 pMoles of mirVana™ miRNA Inhibitor hsa-miR-675-5p (Assay ID: MH12067), or Silencer™ Select Pre-Designed siRNA for long non-coding H19 (siRNA ID: n502891); mirVana™ Negative Control or Silencer™ Select Negative Control were respectively used as negative control (ThermoFisher Scientific). Six hours after transfections, cells were incubated in a hypoxic chamber for 18 h, after which the medium was collected and the cells were processed for following experiments. For miRNA targets’ validation, SW620 and HCT116 cells were seeded at 50.000 cells/cm^2^ in a 12-well plate and transfected for 24 h with 30 pMoles of mirVana hsa-miR-675-5p mimic (Assay ID MC12067) or mirVana™ Negative Control.

### 4.3. RNA Extraction and Real-Time PCR

Total RNA was extracted using Trizol Reagent (cat. number 15596020, ThermoFisher Scientific) according to standard protocol. The concentration of total RNA was detected with a Nanodrop spectrophotometer and 1 μg was used to synthesize cDNA by using High Capacity cDNA Reverse transcription Kit (cat. 4368814, Applied Biosystem; Foster City, CA, USA). QRT-PCR was done in 48-well plates using the Step-One Real-Time PCR system (Applied Biosystem). TaqMan probes were used to evaluate the levels of VEGF (Hs00900055_m1), SNAIL (Hs00195591_m1), and H19 (Hs00399294_g1) using β-actin (Hs00357533) as a housekeeping gene. For microRNAs’ evaluation, 10 ng of RNA were reverse-transcripted by TaqMan MicroRNA Reverse Transcription Kit (cat. number 4366596, Applied Biosystem), and TaqMan^®^ MicroRNA Assays (assay ID: 002005 and assay ID: 001973) were used to amplify has-miR-675-5p and U6 snRNA.

QRT-PCR analyses with Fast SYBR™ Green Master Mix (ThermoFisher Scientific) were carried out to evaluate cyclin D1, c-MYC, PPP2CA, PPP2R1A or PPP2R2B mRNA levels and β-actin was used as a housekeeping gene. The sequences of the primers are reported in Table 1. The fold changes in the mRNA expression level or miRNA levels normalized respectively to β-actin or U6 snRNA housekeeping genes were determined with the ΔΔCt method [65]. The data were expressed as fold of induction (FOI) of indicated mRNA or miRNA after inhibitor transfection compared to relative controls.

### 4.4. TOP/FOP Luciferase Assay

TOP/FOP flash assay was used to evaluate β-catenin TCF/LEF promoter activity. TOP/FOP vectors contain two sets of three copies of the TCF binding site (wild type in TOP and mutated in FOP) upstream of the Thymidine Kinase (TK) minimal promoter and the firefly luciferase open reading frame. FOP Flash was used as a control for measuring nonspecific reporter activation. SW620 and HCT116 cells seeded at 50.000 cell/cm^2^ in a 12-well plate were transfected respectively with 100 ng of TOP or FOP plasmids and then co-transfected with miRNA inhibitor, siH19 or scrambled negative control as described above. For all luciferase assays, pRenilla-CMV luciferase vector was used as an internal transfection control, co-transfected in all conditions at 100 ng/mL. After six hours of transfection, cells were incubated in a hypoxic chamber for 18 h. Lithium chloride (LiCl), a well-established inducer of β-catenin nuclear activity, was added in some samples as a positive control. Finally, the luciferase assay was performed with the Dual-Luciferase Assay System kit (cat. E2920, Promega) and results were detected by GloMax (GloMax-Multi Detection System; Promega). Relative luciferase activity was reported as the fold induction after normalization for transfection efficiency. Lipofectamine 3000 reagent was used for all the described transfections.

### 4.5. GSK-3β and HIF-1α ELISA Assays

Total GSK-3β and phosphorylated GSK-3β were quantified using ELISA GSK-3β (Total/Phospho) Multispecies InstantOne™ ELISA Kit (cat. 85-86173-11, ThermoFisher Scientific) according to the standard protocol. Briefly, cells were lysed with specific lysis buffer provided by the kit to obtain protein extract, 50 μg of proteins were added to the coated 96-well plate and analyzed at 450 nm with Gen5 Microplate Collection and Analysis Software Data.

To detect and quantify HIF-1α transcriptional factor activity, an ELISA assay was performed with ELISA-based kit (TransAM Kit, cat. 47096, Vinci-Biochem, Italy) following manufacturer’s protocol. Briefly, nuclear proteins were extracted by using the Nuclear Extract Kit (Vinci-Biochem) and 8 μg of the samples were added to the coated plate and analyzed at 450 nm with Gen5 Microplate Collection and Analysis Software Data (BioTek Instruments, Inc.^®^). Data were expressed as HIF-1α protein content in total nuclear extract (absorbance).

### 4.6. Western Blot

SDS-PAGE and Western blotting were performed according to standard protocols. SW620 and HCT116 cells were lysed for 1h in lysis buffer containing 15 mM Tris/HCl pH 7.5, 120 mM NaCl, 25 mM KCl, 1 mM EDTA, 0.5% Triton X100, and protease inhibitor cocktail. Cell debris were removed by centrifugation at 17.000 rpm for 20 min at 4 °C. For nuclear/cytoplasm, differential extraction was used with the Nuclear Extract Kit (Vinci-Biochem) following manufacturer’s indications. The protein concentrations were determined by the Bradford microassay method (Sigma; St. Louis, MO, USA) using bovine serum albumin (BSA, Sigma) as a standard. A total of 20 µg protein from each sample was separated using Bolt Bis-Tris gel 4%–12% (ThermoFisher Scientific) and transferred on nitrocellulose membranes (GE Healthcare; Chicago, IL, USA). The membrane was then blocked in 5% BSA solution (5% BSA, 20 mM Tris, 140 mM NaCl, 0.1% Tween-20) and incubated overnight with primary antibodies: anti-β-catenin (1:1000, cat. number sc7963, Santa Cruz Biotechnology; Dallas, TX, USA), anti-histone H1 (1:1500, cat. number 61201, Active Motif^®^, Carlsbad, CA), anti-lamin B (ThermoFisher Scientific), and anti-α-tubulin (1:1000, cat. number sc398103, Santa Cruz Biotechnology). The membrane was incubated with appropriate secondary fluorescent antibody dylight 488 or dylight 594 (ThermoFisher Scientific) and signal was detected by Chemidoc Biorad acquisition instrument. The obtained images were analyzed with the Image Lab software (Bio-Rad; Hercules, CA, USA).

### 4.7. Immunofluorescence Assay

For immunofluorescence, assay cells were fixed in 4% paraformaldehyde, and stained with β-catenin primary antibody (1:100, cat. number sc7963, Santa Cruz Biotechnology). The secondary antibody used was Alexa-Fluor 488 (Life Technologies). Samples were counterstained with Hoechst 3342 (1:1000, Life Technologies) for 10 min at room temperature to detect nuclei, and analyzed by confocal microscopy (Nikon A1) median nuclear planes 

### 4.8. Kaplan-Meier Curves 

The potential effect of miR-675 expression on the overall survival of GC patients was investigated by using Kaplan–Meier plotter online tool (available at https://kmplot.com/analysis/index.php?p=service&cancer=pancancer_mirna). A cohort of 160 rectal cancer patients was selected, and patients were divided into low and high expression groups based on an upper-tertile cut off (cut off value = 20). The analysis was not restricted to a certain tumor stage, gender or race. Survival threshold was set as “all”, i.e., no patients were excluded or censored from the generation of the plot, no matter how long they survived. The Compute median survival box was checked, and so was the Censored at threshold box, but the last one had no effect on the plot computation, because no specific threshold was selected.

### 4.9. mirWalk Target Prediction and Pathway Mapping

A target prediction and then a pathway mapping of the putative target genes was performed by using the mirWalk 2.0 [32] database search engine, specifically, the Predicted Target > MicroRNA-Gene Target module, available at http://zmf.umm.uni-heidelberg.de/apps/zmf/mirwalk2/miRretsys-self.html. The miRbase identifier hsa-miR-675-5p was used as a query, with all the others parameters set as default. The reference database for pathway mapping was KEGG. 

### 4.10. Statistical Analysis

The data obtained was derived from the mean of at least three experiments, giving reproducible results, and are represented as the mean ± standard deviation (SD). For statistical analysis, two-tailed non-parametric Student’s *t*-test was performed using GraphPad Software Inc. The statistical significance, if it is present, is reported as *p*-value in the figures. 

## 5. Conclusions

The presented data highlighted a new molecular mediator in the crosstalk between HIF1 and β-catenin. These results, together with our previous studies demonstrating that miR-675-5p expression enforces the hypoxia-induced EMT in CRC [14], make the miR-675-5p a new interesting target to inhibit both CRC growth and progression. 

## Figures and Tables

**Table 1:** Sequences of qRT-PCR primers.

Gene	Primer Forward	Primer Reverse
*β-actin*	TCCCTTGCCATCCTAAAGCCACC	CTGGGGCCATTCTCCTTAGAGAGAAG
*Cyclin D1*	AAAGAATTTGCACCCCGCTG	GACAGACAAAGCGTCCCTCA
*c-Myc*	TACAACACCCGAGCAAGGAC	CTAACGTTGAGGGGCATCGT
*PPP2CA*	ACTCGACTCCTGGGCTTTTG	AAACCGTCCCTGACGATGAC
*PPP2R1A*	TGCTCATAGACGAACTCCGC	ACTTCGGGTCCTTTCAACCC
*PPP2R2B*	CAAGGAAAGGGCACATCAACC	GCTCTCTTTCTGTCCCCTGAA

## Supplementary Materials

The following are available online at https://www.mdpi.com/1422-0067/21/11/3832/s1.
