# Supplementary material for: Hypoxia-Induced miR-675-5p Supports β-Catenin Nuclear Localization by Regulating GSK3-β Activity in Colorectal Cancer Cell Lines
Source: Int J Mol Sci. 2020 May 28;21(11):3832. doi: 10.3390/ijms21113832 (PMC7312749; doi:10.3390/ijms21113832)
Supplement: Supplementary file 1 [file ijms-21-03832-s001.pdf]

Table S1. The whole mirWalk2.0 output table, in which predicted miR-675-5p target genes are grouped into known KEGG pathways. Green background marks the significant pathways after p-value adjustment (multiple testing method or FDR correction, BH < 0.05) and yellow background highlights the results that are interesting for this study. The first result is a group of cancer pathways, with a strong significance score, provided by Fisher's correlation test. Other significant pathways are, as expected, Wnt signaling cascade and colorectal cancer-specific pathways. According to the mirWalk site, "PathFg", "PathBg", "GenomeFG" and "GenomeBG" stand for "number of genes predicted as putative targets in a given pathway", "number of genes in a given pathway", "total number of target genes within genome" and "total number of genes within genome". BH values are calculated by correcting the p-value in order to take account for false discovery rates. This table is supposed to serve as a guide to discover predicted patterns that deserve further experimental investigation and should not be considered a validation. In this case, our interest is in Wnt and cancer pathways, and both seem to be significantly correlated with miR-675 activity.

Path Name	PathFg	PathBg	GenomeFG	GenomeBG	Fisher	BH
Pathways in cancer	194	330	7824	19747	1.08767115359224e-12	2.11008203796895e-10
Endocytosis	119	187	7824	19747	2.36849767596898e-11	4.57120051462014e-09
Axon guidance	82	129	7824	19747	3.10261420697335e-08	5.95701927738883e-06
Wnt signaling pathway	92	152	7824	19747	1.41756058999002e-07	2.70754072688093e-05
Calcium signaling pathway	103	178	7824	19747	6.01459913338398e-07	0.000114277383534296
Neurotrophin signaling pathway	78	129	7824	19747	1.30173227685124e-06	0.000244725668048034
Chronic myeloid leukemia	50	75	7824	19747	1.92275693712056e-06	0.000359555547241545
MAPK signaling pathway	145	272	7824	19747	2.95074892577114e-06	0.000548839300193432
Pancreatic cancer	49	75	7824	19747	5.81490004869364e-06	0.00106994160895963
mTOR signaling pathway	37	53	7824	19747	8.18331849790223e-06	0.00149754728511611
Colorectal cancer	54	86	7824	19747	1.14972069760866e-05	0.00210398887662385
Insulin signaling pathway	80	139	7824	19747	1.36945480429449e-05	0.00249240774381597
Glioma	42	65	7824	19747	3.96095474374029e-05	0.00709010899129512
Long-term potentiation	45	71	7824	19747	4.32844611495722e-05	0.00770463408462384
ErbB signaling pathway	54	89	7824	19747	4.66335531297931e-05	0.00830077245710316
Oocyte meiosis	65	112	7824	19747	5.9775120347932e-05	0.010520421181236
Dilated cardiomyopathy	55	94	7824	19747	0.000159516203595992	0.0269582384077227
Chemokine signaling pathway	99	189	7824	19747	0.000241921533764226	0.0404008961386258
Prostate cancer	52	89	7824	19747	0.000250229905150344	0.0417883941601075
Apoptosis	51	87	7824	19747	0.000255407139293556	0.0426529922620238
T cell receptor signaling pathway	62	110	7824	19747	0.000272138442226606	0.0451749814096166
B cell receptor signaling pathway	45	75	7824	19747	0.00027986681181092	0.0464578907606126
Gap junction	52	90	7824	19747	0.000365892436877147	0.0603722520847293
Non-small cell lung cancer	34	54	7824	19747	0.000437412608745171	0.071735667834208
Fc gamma R mediated phagocytosis	55	97	7824	19747	0.000481131819360118	0.0780584942304217
Melanogenesis	57	102	7824	19747	0.00063087954266114	0.100309847283121
Renal cell carcinoma	42	71	7824	19747	0.000669780333302486	0.105825292661793
Focal adhesion	103	203	7824	19747	0.000813095542915883	0.126842904694878
GnRH signaling pathway	58	105	7824	19747	0.000839052621275231	0.130892208918936
Small cell lung cancer	48	84	7824	19747	0.00084939212374525	0.132505171304259
Endometrial cancer	32	52	7824	19747	0.00113140993192025	0.174237129515718
Regulation of actin cytoskeleton	106	212	7824	19747	0.00131980798634701	0.201930621911093

SNARE interactions in vesicular transport	25	39	7824	19747	0.00170957080575779	0.259854762475183
Arrhythmogenic right ventricular cardiomyopathy ARVC	42	74	7824	19747	0.002085871071415	0.314966531783665
Acute myeloid leukemia	34	58	7824	19747	0.00261171058777866	0.3917565881668
Melanoma	40	71	7824	19747	0.00316162631320975	0.471082320668253
Progesterone mediated oocyte maturation	48	88	7824	19747	0.00318490384134943	0.474550672361065
VEGF signaling pathway	43	78	7824	19747	0.0039217479844374	0.569647965044282
Basal cell carcinoma	32	55	7824	19747	0.00405523817315612	0.588009535107637
Notch signaling pathway	28	47	7824	19747	0.00442162446433151	0.636713922863737
Vascular smooth muscle contraction	60	116	7824	19747	0.00535381834849955	0.760242205486936
Hedgehog signaling pathway	32	56	7824	19747	0.0059050954222041	0.826713359108574
Long-term depression	40	73	7824	19747	0.00608159040927497	0.851422657298496
Hypertrophic cardiomyopathy HCM	46	86	7824	19747	0.00625098766282358	0.875138272795301
Aldosterone regulated sodium reabsorption	25	42	7824	19747	0.0071011523180801	0.979959019895054
Circadian rhythm mammal	10	13	7824	19747	0.00718329701506948	0.984111691064519
Inositol phosphate metabolism	28	54	7824	19747	0.0457035400640318	1
Phosphatidylinositol signaling system	40	76	7824	19747	0.0144558256057898	1
Lysosome	59	121	7824	19747	0.0253264686283214	1
Biosynthesis of unsaturated fatty acids	13	22	7824	19747	0.0509852902103153	1
p53 signaling pathway	34	68	7824	19747	0.0528094460844275	1
Ubiquitin mediated proteolysis	64	134	7824	19747	0.0334039905907191	1
Pentose phosphate pathway	17	27	7824	19747	0.0119823127887169	1
Purine metabolism	73	158	7824	19747	0.0537983179526431	1
Dorso ventral axis formation	14	24	7824	19747	0.0492932162866022	1
Cell adhesion molecules CAMs	62	133	7824	19747	0.0595170796154884	1
Primary bile acid biosynthesis	10	16	7824	19747	0.0546237774699997	1
Amyotrophic lateral sclerosis ALS	30	55	7824	19747	0.0175786299345459	1
Thyroid cancer	17	29	7824	19747	0.0297814820374443	1
Adipocytokine signaling pathway	37	70	7824	19747	0.0167841882148267	1
Tight junction	62	132	7824	19747	0.0510986135923944	1
Adherens junction	39	76	7824	19747	0.0253272083114254	1
Bladder cancer	23	43	7824	19747	0.0453807292108869	1

Table S2. mirWalk Analysis. MirWalk [26] identified consensus sequence for the miR-675-5p (miRbase MIMAT0004284) on the transcripts indicated in the table.

genesymbol	refseqid	start	end	energy	number_of_pairings	binding_region_length	longest_consecutive_pairings	position
GSK3B	NM_002093	4126	4151	-25.9	19	25	10	3UTR
	NM_002093	1965	1998	-26.3	21	33	7	CDS
	NM_001146156	4087	4112	-25.9	19	25	10	3UTR
	NM_001146156	1926	1959	-26.3	21	33	7	CDS
PPP2CA	NM_002715	2209	2248	-27.8	20	25	10	3UTR
PPP2R2B	NM_181674	205	265	-28.9	22	48	5	5UTR
	NM_181675	1302	1334	-24.8	19	32	7	CDS
	NM_181675	167	195	-25.7	18	28	8	CDS
	NM_181676	205	265	-28.9	22	48	5	5UTR
	NM_181678	1225	1257	-24.8	19	32	7	CDS
	NM_001271899	1255	1287	-24.8	19	32	7	CDS
	NM_001271900	1449	1481	-24.8	19	32	7	CDS
	NM_001271948	167	195	-25.7	18	28	8	5UTR
PPP2R1A	NM_014225	1003	1045	-32.4	19	34	10	CDS
	NM_014225	696	731	-31.7	19	35	11	CDS
	NM_014225	2136	2162	-28.2	20	26	8	3UTR

Supplementary Figure S1

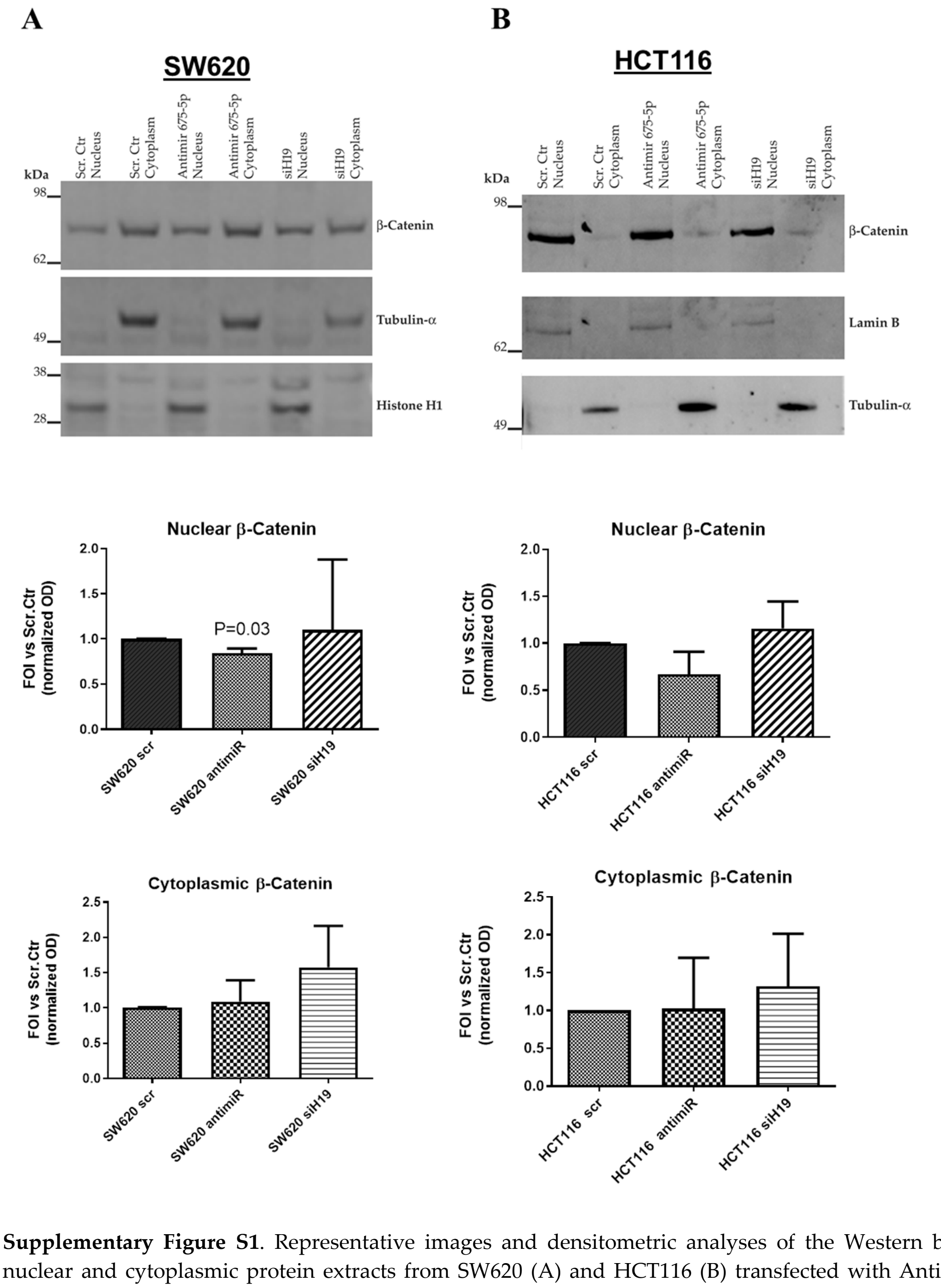

Supplementary Figure S1. Representative images and densitometric analyses of the Western blots for β-catenin on nuclear and cytoplasmic protein extracts from SW620 (A) and HCT116 (B) transfected with AntimiR-675-5p, siH19 or scrambled negative control and subjected to 18 hours of hypoxic stimulation.

Supplementary Figure S2

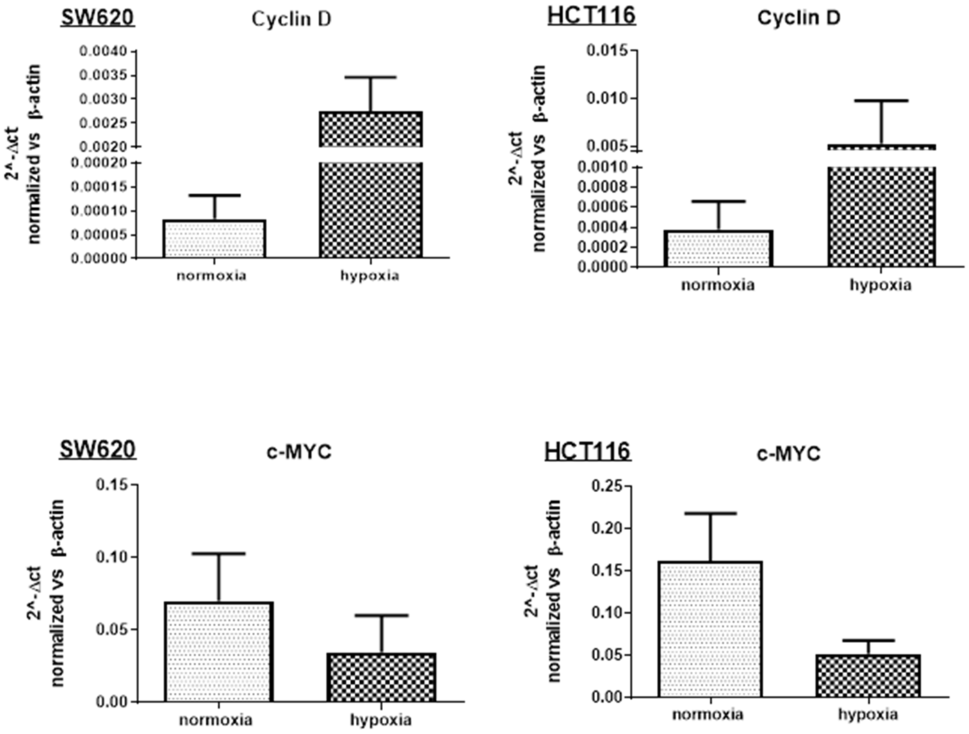

Supplementary Figure S2. Real-time PCR for c-MYC and Cyclin D1 in SW620 (left panels) and HCT116 (right panels) cells in normoxic conditions subjected to 18 hours of hypoxic stimulation. Gene expression data represented as 2^-Δct normalized for β-actin. Data are expressed as the mean ± SD of three independent experiments.
